# Tino Rangatiratanga and Well-being: Māori Self Determination in the Face of Covid-19

**DOI:** 10.3389/fsoc.2021.613340

**Published:** 2021-02-03

**Authors:** Annie Te One, Carrie Clifford

**Affiliations:** ^1^ Victoria University of Wellington Te Herenga Waka, Wellington, New Zealand; ^2^ University of Otago, Dunedin, New Zealand

**Keywords:** tikanga, health, COVID-19, self determination, tino rangatiratanga, hauora, indigenous health, Māori

## Abstract

The New Zealand government has been globally praised for its response to Covid-19. Despite the global accolades, little attention has been given to the swift and innovative Māori response to Covid-19. This paper will detail some of this rapid Māori response to Covid-19 in Aotearoa New Zealand and argue the response can be understood as key examples of Māori exercising tino rangatiratanga (self-determination), independent of the government’s measures and policies. We suggest that this exploration of tino rangatiratanga during Covid-19 demonstrates central aspects of Māori well-being that move beyond a government focus on statistics as the key measure of well-being and how tikanga Māori (Māori values) are being used to develop successful responses to the global pandemic.

## Introduction

Like many people and places around the world, 2020 has required significant changes to the way we work, socialise and carry out our day to day lives due to the ongoing impacts of Covid-19. Indigenous communities from around the world have had to make significant adaptations to cultural practices in order to protect the health and well-being of their communities. This article discusses some of the actions that Māori the Indigenous peoples in Aotearoa New Zealand have taken to protect their communities, while also maintaining and promoting tino rangatiratanga (self-determination).

In March 2020, with only 183 cases of Covid-19 the New Zealand government moved the entire country into a lockdown under level four restrictions. The decisive action taken by the New Zealand government has been heralded around the world as one of the most effective solutions to limiting the reach and impact of Covid-19. Since the reduction in alert levels, there has been a small swell in cases, but most are traced and controlled, with social distancing measures in place across the country. The measures taken by the New Zealand government has meant that the devastation that has been caused by Covid-19 throughout the world, has not been felt in the same way in our country.

A number of Māori communities throughout Aotearoa, also took swift and rapid action to the pandemic, which in many ways built on and exceeded actions taken by the New Zealand government.

This article explores those actions and the ways in which there is a cyclical and inter-dependent link between tino rangatiratanga and well-being. In this article, the authors argue that tino rangatiratanga enables positive well-being, and equally, the positive well-being of individuals and communities enables the exercise of tino rangatiratanga. In other words, despite the impacts of colonisation, Māori communities have maintained and built strong levels of tino rangatiratanga, to both protect and enable tino rangatiratanga to thrive. We suggest that an important way of understanding the actions taken by certain Māori communities, is through an analysis of tikanga Māori (Māori values), which provide an Indigenous lens for understanding partially why and how communities responded as they have to Covid-19.

This article will begin by providing a contextual discussion of the New Zealand governments responses, before a brief on the importance of intergenerational storytelling. This is followed by a brief analysis and explanation of tino rangatiratanga, tikanga (Māori values) and hauora, before discussing five examples of Māori responses to Covid-19. Finally, we make some recommendations which we hope will provide a basis for how governments and Indigenous peoples alike can strengthen responses to Covid-19 while also supporting well-being and Indigenous self-determination. Responses to Covid-19 have not only shown the strength of Māori leadership, but more broadly Indigenous leadership, and the potential for Indigenous-led solutions, such as a values-driven holistic health approach. This could positively shape the global Covid-19 responses and impact the health of all.

### Contextual Information - Overview of Covid-19 in Aotearoa (New Zealand)

**Figure 1:**
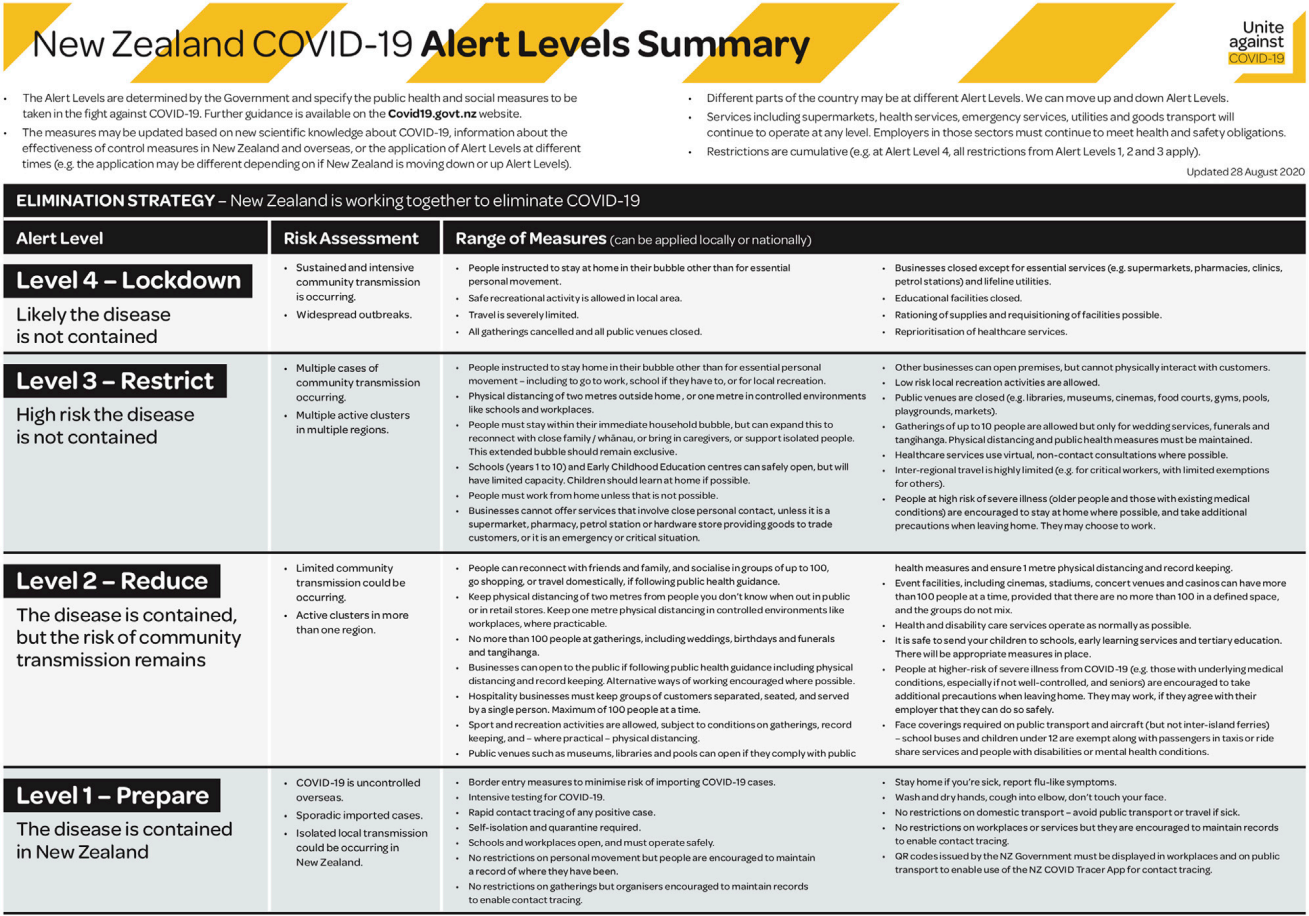
New Zealand COVID-19 alert levels summary. Source. New Zealand Government (2020).

The first confirmed case of Covid-19 in New Zealand was reported on the February 28, 2020, related to international travel. As the number of cases started to increase the strategy of the New Zealand government was to go hard, and go early (Jamieson, 2020). On the 19th of March 2020, for the first time in history, the government closed the country's borders to all but New Zealand citizens and permanent residents. By March 25th, the New Zealand government declared a state of emergency and the entire nation moved into Alert Level 4 Lockdown (see Figure 1 for an Alert level summary) for a minimum of four weeks, with the exception of essential workers. Prime Minister Jacinda Ardern stated, *“We have a window of opportunity to stay home, break the chain of transmission, and save lives,”* (Strongman, 2020, p. 20, p. 20)*.* This lockdown was extended by five days, and New Zealand moved to Alert level 3, Tuesday 28 April. As case numbers continued to remain stable and community transmission was ruled out, New Zealand moved down alert levels (New Zealand Ministry of Health, 2020). Aiding the response efforts, geographically, New Zealand is relatively isolated from the rest of the world, and New Zealand also got its first case relatively late in the outbreak - this giving us time to prepare and other responses to model off. As international travel is the main source of entry of Covid-19, New Zealand established strict government-sanctioned managed isolation facilities to quarantine those who enter the country, these still remain today.

Two days after reaching the milestone of no community transmission for 100 days, a milestone reached by very few countries, on the ninth of August, New Zealand confirmed its first Covid-19 case associated with community transmission, and there has been a resurgence located in the Auckland area. As of the 20th of September, there have been 1,464 confirmed cases of Covid-19 in New Zealand (with 351 probable cases), making the total number of confirmed and probable cases 1,815. Sadly, there have been 25 deaths related to Covid-19, with 71 active cases remaining (New Zealand Ministry of Health, 2020). Of relevance, to date, Māori make up 10% of total Covid-19 cases, yet Māori make up approximately 16.5 percent of the New Zealand population (Statistics New Zealand, 2019). Given this context, we now discuss the Maori response to Covid-19, beginning with a discussion on tino rangatiratanga (self-determination).

### Tino Rangatiratanga

Tino rangatiratanga can mean self-determination, sovereignty, independence, autonomy. The term itself is rooted in a Māori worldview, and there is no one English term which fully encapsulates its meaning. Tino rangatiratanga refers to Māori control over Māori lives, and the centrality of mātauranga Māori (Māori knowledge). While focused on a Māori worldview, tino rangatiratanga also has a close association with the challenges that have come from the loss of Māori control through colonial practices, and has been used as a framework from which Māori have continued to challenge governments for recognition of our individual and collective self-determination.

The term “rangatira” makes up the term, and is the Māori word for leader, again this English term does little to inform about the worldview through which Māori leadership is understood. The term can be broken into two parts “ranga” and “tira.” Ranga derives from the term “raranga” which means to weave, and “tira” refers to a group or community. In essence, leadership from a Māori perspective, is dependent upon a leader’s ability to weave a community together, while recognising the different strands which exist within a group (Mikaere, 2010). For Māori therefore, issues and acts of self-determination refer to the collective well-being of a group, for Māori these social ties include iwi (tribes), hapū (sub-tribes) and whānau (families), all of which form the main sites through which Māori tino rangatiratanga is claimed and exercised.

In some cases, actions toward tino rangatiratanga has involved overt and direct challenges to the Crown, such as protests, hīkoi (protest marches), sit ins, or utilising legal avenues, all tools through which Māori have sought to maintain and regain some level of tino rangatiratanga (Harris, 2007). However, tino rangatiratanga, and indeed indigenous self-determination can be exercised through diverse and multiple avenues. First nations scholar Jeff Corntassel (2012) for example has discussed at length how indigenous self-determination should not always be reactionary or enacted only in response to the Crown. Corntassel argues that self-determination can and is strengthened the most by indigenous peoples practicing indigenous ways of being, irrespective of a need to gain rights or recognition from the Crown. In a similar way, Māori academic Maria Bargh (2013) has argued that Māori politics occurs through “micro-oriented” practices, in which Māori practice political authority in small and diverse ways, such as establishing local, iwi gardens to support food sovereignty. These ideas relate directly to tino rangatiratanga in that Māori continue to actively promote Māori ways of being that are not only directed at reclaiming rights from the Crown.

The Māori responses to Covid-19 demonstrate numerous ways through which Māori expressed tino rangatiratanga directed at protecting and supporting local communities that happened irrespective of the actions being taken by the New Zealand government. These actions show how tino rangatiratanga is enacted as independent Māori decision-making. We suggest that this independent decision-making and indeed the exercise of tino rangatiratanga was partially made possible through adherence and respect of tikanga Māori.

### Tikanga Māori

Tikanga are the rules and values that are founded within a Māori worldview. According to Mead (2016) ‘Tikanga Māori might be described as Māori philosophy in practice and as the practical face of Māori knowledge’ (p. 15). As such, tikanga are understood to cover all aspects of life and facilitate a range of relationships between individuals and groups that extends throughout generations.

Tikanga are also often viewed as a Māori legal framework, but as Jones (2016) has discussed at length, there are some key distinctions, which in particular are about the adaptability and flexibility of tikanga in relation to the rigidity of western law. Tikanga Māori are diverse and flexible by nature. In some ways, the strength of tikanga is that they are made to adapt to differing circumstances and changes to environments and contexts. Indeed, Māori have continued to develop tikanga for generations in order to address the changes that have occurred on our lands. However, while adaptability is a strength of tikanga, there are numerous identifiable values which provide guidance about appropriate measures and actions. In this way, tikanga provides guidance on how people should act, as well as why people should act. In other words, tikanga confirm the responsibilities that Māori have to one another as a basis for action.

While it is beyond the scope of this article to flesh out the numerous different tikanga within a Māori worldview, we suggest that part of how tino rangatiratanga was exercised during the Covid-19 response was through Māori drawing guidance and responsibilities from tikanga Māori. Some of the tikanga that we illustrate in further depth in the case studies below, include; *Mana* – this is power, authority, or prestige and can be understood also as Māori political authority and control. Both individuals and groups can exercise mana, and for an iwi and hapū to be recognised as the authority over their lands and resources, it is considered that they have mana (Smith, 2013; Mead, 2016). The examples discussed below demonstrate how iwi and hapū have maintained their mana through the political decision-making and actions that were taken.

*Manaakitanga* – this relates to care and respect. Essentially, if mana is to be maintained, then there need to be efforts in place which demonstrate reciprocal care between people and groups (Smith, 2013; Mead, 2016). Again, manaakitanga can be used to understand the actions taken by Māori during the Covid-19 pandemic to protect and provide for communities.

*Kaitiakitanga* – this is care and guardianship over the environment and relates to the genealogical connections that Māori claim to the natural environment (Smith, 2013). In essence, Māori must protect the land in return for living off the land (Mead, 2016). For some iwi, their decisions regarding community protection during the Covid-19 restrictions were influenced heavily by the need to act as kaitiaki (guardians) over their lands.

*Whanaungatanga*- this is relationships, both familial and non-familial. For Māori relationships between people are required in order for society to operate (Smith, 2013; Mead, 2016). Whanaungatanga requires purposeful efforts to seek out and create lasting relationships as a mechanism to support individuals and establish collective ties. For this reason, understanding the Covid-19 response as articulated through tikanga, provides context for why Māori took the actions to maintain strong community links.

This is not a finite list of tikanga by any means, nor a full explanation of the tikanga that have underpinned Māori responses to Covid-19. However, understanding Māori responses and indeed the various ways that tino rangatiratanga can be enacted through a Māori values system could provide a basis through which Māori leadership in hauora and well-being can be understood. Furthermore, using a tikanga lens to assess how tino rangatiratanga and well-being intertwine demonstrates how one of the main strengths of tikanga is the ability to rapidly adapt even in the most unprecedented circumstances.

### Hauora - Māori Well-Being

Hauora refers to the holistic Māori philosophy of health and well-being. Hauora consists of two words, hau and ora. Hau translating to breath and ora meaning to be well, and together they refer to the breath of life. Heaton (2018) explains that his combination of terms has roots in Māori creation narratives where the first woman Hine-ahu-mai-i te-one was given the first breath of human life from a Māori god Tāne. Hau (wind or vital essence of life), ha (breath), ora (to be alive, healthy, to survive) and wairua (spirit) were infused into the first female, who holds the ability to create life (Heaton, 2018).

Māori are not a homogenous group, and while notions of well-being vary according to whanau, hapū and iwi, there are some common elements. A Māori worldview of health is holistic in nature and extends far beyond a biomedical model of health which focuses on purely biological factors and excludes psychological, environmental, and social influence (Durie, 1998). Whānau is the foundation of Māori society, and as a principal source of strength, support, security and identity, whānau plays a central role in the well-being of most Māori, both individually and collectively. In a Māori worldview, each whānau member, (from young children to elders), is valued and plays an integral part in contributing to the well-being of the overall whānau (New Zealand Ministry of Health, 2002).

Māori health frameworks have been created in an attempt to help articulate important aspects of hauora. Professor Mason Durie’s (1998) Te Whare Tapa Wha model compares hauora to the four walls of a whare (Māori meeting house), each wall representing a different dimension of hauora: taha wairua (spiritual well-being); taha hinengaro (mental and emotional well-being); taha tinana (physical well-being); and taha whānau (family and social well-being) which includes collective aspects of well-being. In this model, each of these four dimensions of hauora influences and supports the others and are necessary for strength, symmetry and balance. In addition to these dimensions of hauora outlined by Durie, in Rangimārie Rose Pere's (1991) model of well-being - Te Wheke - Pere also identifies Mauri (life force in people and objects), Mana ake (the unique identity of individuals and family), Hā a koro ma, a kui ma (the breath of life from forbearers), and Whatumanawa (the open and healthy expression of emotion), as important contributors to whānau health. This model also highlights the important interconnectedness of all of these factors (Pere, 1991). Mauriora (cultural identity), Waiora (connection with the physical environment), Toiora (healthy lifestyles), Ngā Manukura (leadership), Te Mana Whakahaere (autonomy at a community level) and Te Oranga (participation in society) are also important aspects of Māori well-being - this outlines in the Te Pae Māhutonga public health framework (Durie, 1999). While these models do vary in how they conceptualise well-being, they all highlight that Māori understandings of well-being must be understood in a holistic, collective manner and one that includes supporting collective ties. While an analysis of these models is outside the scope of this article, we suggest that these understandings of hauora which center on holistic and collective well-being are essential parts of both why and how Māori responded to Covid-19. Furthermore, these models which are bound in a Māori worldview, relate directly to the tikanga that we have provided above.

### Linking Tino Rangatiratanga and Māori Well-Being

The understanding that tino rangatiratanga and Māori well-being is linked is briefly alluded to in pre-existing literature, however in little detail (Panelli and Tipa, 2007; Cram, 2014; Jackson et al., 2018). Examining the Māori response to Covid-19 through a dual tikanga-well-being lens allows us to see how they are linked (in a real-life health context) which builds a better understanding of how this complex relationship plays out and why it is so important moving forward.

Enacting tino rangatiratanga is essential to achieve Māori individual and collective well-being. However, Māori well-being is also a foundation of Māori development (Cram, 2014) therefore, a certain level of well-being of a peoples - including their culture and language - is needed to enact tino rangatiratanga. As a result, tino rangatiratanga can be viewed as both a marker of, as well as an important contributor to well-being. And equally, well-being is an important contributor to tino rangatiratanga. The interconnected and reciprocal relationships between tino rangatiratanga and well-being can be seen during the Covid-19 response, where the cultural values and practices, as well as the capacity of Māori people, contribute to the enacting of Tino Rangatiratanga, in turn, protected and promoted the well-being for all New Zealanders.

Māori did not just enact tino rangatiratanga in response to Covid-19. Generations of language and cultural revitalisation efforts, allowed for matauranga Māori and the use of tikanga; the capacity building of Māori academics, researchers, and health professions (as previously mentioned), over the last 30 years allowed for a swift, culturally tailored, Māori health response, as well as an acute awareness of factors that could lead disparate health outcomes (such as pre-existing health conditions, as well as documented racism in the health care system); Treaty of Waitangi claims and the organisation of hapū and iwi organisations allowed for economic self-determination and a coordinated care response. While significant struggles still exist for Māori, we have continued to reaffirm our tino rangatiratanga in diverse ways, which have helped us to respond proactively to Covid-19.

### Case Studies

The following section provides five case studies of how Māori responded to Covid-19. We clearly demonstrate the link between tino rangatiratanga and well-being and how the responses were informed by tikanga Māori.

### Māori Storytelling Practices Guide Covid-19 Response

Oral traditions and intergenerational storytelling practices are a fundamental part of Māori culture and communities and played an important role in the rapid Covid-19 response by Māori communities. Māori cultural practice of remembering our ancestors - their lives, experiences, characteristics, their passing - meant that Māori were acutely aware of the potentially devastating impacts that Covid-19 could have on their communities. Throughout history, epidemics and introduced diseases have had a devastating impact on Māori communities. European colonisers brought new diseases such as measles and flu to New Zealand and because Māori lacked natural immunity to these diseases, many died. From the first European contact to 1840, Māori lost an estimated 30% of our entire population, mostly to epidemics (Lange, 2011). A further 30 per cent were lost in the twenty years that followed. The influenza epidemic of 1854 killed over 5,000 Māori, and the Māori death rate from the 1918 Spanish flu, was more than eight times that for non-Māori (New Zealand Ministry for Culture and Heritage, 2020; Ngata, 2020).

Today, Māori continue to tell stories about the impact these infectious diseases had on their population, language, culture and community. Māori immortalise these stories in physical reminders, such as pictures and carved pou, to ensure they speak about and remember the devastating disproportionate impact epidemic’s such as the Spanish Flu had on Māori communities. This is deliberate “We keep our ancestors close, their memories live on with us, and their lives become our lessons” (Ngata, 2020, p.1). Because of this way of remembering past events, Māori were accurately aware of the impact previous pandemics and introduced diseases had on our communities and the potential impact Covid-19 could have. This promoted Māori to react quickly and purposely, with knowledge, innovation, insight and awareness of consequences, which built the foundation for tino rangatiratanga to take place. The foundation of the Covid-19 response was built upon this important well-being practice.

### Iwi Checkpoints

Iwi checkpoints were perhaps the most well-known action undertaken by iwi throughout the country. As the Covid-19 threat became ever more present in Aotearoa, a number of iwi took it upon themselves to protect local communities by establishing monitored entry and exits from their communities. These actions complimented decisions made by the New Zealand government, who had made nationwide rules to restrict movement, however as the history of pandemic devastation in Māori communities is still being felt, this extra measure taken by Māori communities sought to provide an extra layer of protection. These checkpoints saw iwi members organising to stop travellers from entering their communities- unless they were residents- to avoid any risk of spreading Covid-19. The iwi checkpoints were essentially about protecting the well-being of entire communities, Māori and non-Māori, and represent clear sites of iwi exercising tino rangatiratanga. The communities where such checkpoints were established were towns with high Māori populations, As such, some iwi rūnanga (tribal organisations) effectively mobilised in ways that demonstrated a capacity to protect well-being due to strong systems of governance that were already in place. Iwi such as those in Te Tai Tokerau, Te Whānau a Apanui, Tūhoe and Taranaki iwi all set up various checkpoints which were guided by both a need to protect the health of communities, but also responses that were driven through tikanga. Numerous media reports covering the iwi checkpoints and interviews with iwi leaders throughout the country demonstrates the centrality of tikanga and rangatiratanga in informing the rapid responses. Co-ordinator of the Northland iwi checkpoints for example stated that their decision to close off communities was based on their position as kaitiaki (guardians) which was supported by iwi rangatiratanga; “If we have this kaitiaki status and our own rangatiratanga, we have to step up and participate in serious issues like this,” (Taipari, cited in de Graaf, 2020b).

Other iwi such as Tūhoe also confirmed through media interviews that their decision to control movement in and out of their community was based on their position as kaitiaki and the obligations and responsibilities they had to protect local communities; “Our role as kaitiaki in this case means keeping people safe and ensuring this closure is respected.” (Kruger cited in Williams and Biddle, 2020).

Essentially these decisions and those made in other parts of the country can be linked in through the responsibilities that iwi have and are driven through tikanga Māori. For example, these actions align succinctly with kaitiakitanga (guardianship) and the responsibility that iwi have to protect the health of their environments, which is inseparable from the health of their people. In turn, these kaitiaki responsibilities are also driven through manaakitanga (caring, nurture) and that care for communities is essential and could be practically protected through monitoring travel in and out of communities. These tikanga also come hand in hand with the exercise of mana (authority, power), in that iwi mana is reconfirmed through actions that are taken to support the health of a community. These instances demonstrate the continued importance of whanaungatanga, in that social relationships are embedded in these communities as being essential to the way that iwi operate. Protecting relationships and utilising those iwi relationships to develop a planned and coordinated response was central to iwi checkpoints. Understood through a tikanga lens provides important lessons on how Aotearoa could develop well-being frameworks and structures that are underpinned by tikanga Māori. Many other Indigenous communities, such as Aboriginal Australians also identified themselves as being at risk of infection and death from COVID-19. They also enacted sovereign actions and moved quickly to keep COVID-19 out of their communities in remote regions by restricting access to both outsiders and returning Aboriginal community members (Schultz, 2020).

Iwi checkpoints provide important examples of how current iwi governance structures are in a position to mobilise in situations that require rapid responses. These are clear examples of how iwi understand their tino rangatiratanga and that an essential part of maintaining tino rangatiratanga is to protect and promote the well-being of local communities.

### Kōhanga Reo

A lesser publicised example, which equally demonstrates Māori enacting tino rangatiratanga during the Covid-19 crisis, was the decision made by Te Kōhanga Reo National Trust advising all kōhanga reo (Māori language preschool’s) to remain closed during alert level 3, this despite the New Zealand Government lifting restrictions and recommended early childhood programmes could go back at full capacity.

While the New Zealand Government had announced that Education providers and children could return to schools, Te Kōhanga Reo National Trust made an independent decision for children to remain at home. This decision was made as a matter of caution and protection for Māori communities beyond the national standards. Trust chief executive, Angus Hartley stated, “The Trust believes whānau should take an extra precautionary approach and not risk the health and well-being of our vulnerable pakeke (adults), kaumātua (elders) and mokopuna (grandchildren)”. “Further, stating that the”. The advice from the ministries of education and health is not consistent with the risk profile that exists at kōhanga reo” (Hartley, cited in Hurihanganui, 2020, p.1). This refers to the number of kaumātua that work in kōhanga reo. In enacting their own tino rangatiratanga the Trust surveyed their kōhanga reo whānau and found that over a third fall into high-risk groups and were therefore at a heightened risk to Covid-19. Furthermore, nearly 80% of those associated with kōhanga reo reported that they did not feel safe to return to mahi (work) at alert level 3 (Hurihanganui, 2020).

This extra precautionary approach demonstrates Māori decision making independent of Government and the ability to identify and manage risk unique to their own communities. As described above, in relationship to the checkpoints, these actions align with kaitiakitanga and the responsibility that Māori organisations, such as Kōhanga Reo, felt to protect the health of their whānau, employees, as well as the children they care for. In turn, these kaitiaki responsibilities also expressed manaakitanga for their most vulnerable as well as the importance of whanaungatanga and how responses to Covid-19 require consultation with communities and those groups that connect into decisions being made. In this case study, Māori made their own decisions based on a process of observation and understanding their community, as well as the current circumstances.

### Care Packages

Many hapū, iwi, Māori organisations and community groups organised and delivered care packages to members of the community. Priority was given to vulnerable members of the community, such as the elderly, low-income earners, as well as those who live rurally, or had pre-existing health conditions. While there is no official reporting of the exact number and type of resources distributed by iwi, hapū and Māori organisations, it was on the largest scale seen in recent history. For example, the iwi Ngā Puhi and Waikato Tainui distributed 8,000 and 5,000 food packages respectively (de Graaf, 2020a) and Te Pūtahitanga - the Whānau Ora commissioning agency for Te Waipounamu (South Island of New Zealand) distributed 1,734 food packages. Te Pūtahitanga also distributed 1,371 grants for home heating, 1,104 data support, 600 devices to enable digital connectivity and 25,000 hygiene packages (McMeeking and Savage, 2020).

Whānau Ora commissioning agencies were recognised as key to increasing outreach to Māori communities and ensuring equitable and holistic care. The New Zealand Ministry of Health provided $4.3 million to the Whānau Ora commissioning agencies to support their work. A direct result of this funding was the distribution of 80,000 hygiene packages to whānau across Aotearoa, ensuring those in need had appropriate cleaning and hygiene materials. Whānau Ora also provided over 2,500 grants directly to whānau to support them, averaging over $400 per whānau. Additionally, 7,898 whānau received Manaaki Support packages, which included food, data support, and other material means of support via Whānau Ora Navigators (New Zealand Ministry of Health, 2020). Looking globally, Native American grassroots community members demonstrated a similar response, also providing food to vulnerable community members, in one case filling the void left by canceled feeding programs, which would have disproportionately impacted the elderly and youth (Hoover, 2020).

Care packages demonstrate a holistic Māori health response which extends beyond a physical health response to ensure that Māori, in particular our vulnerable community members, were taken care of during Covid-19 lockdown, this also ensuring that existing hardships were not perpetuated during this time. The large numbers of packages provided from across agencies is significant as it demonstrated ways in which individual iwi, hapū and Māori organisations understood the key supports that were needed. This response was based on the import value of manaakitanga and also exemplifies the importance of whanaungatanga, and existing trusted relationships with Māori communities was key to successful outreach to Māori communities and ensuring equitable and holistic care.

### Online Innovations

Understanding the use and development of online forums through a tikanga lens, highlights the adaptability of tikanga to maintain whanaungatanga. Relationships are central to tino rangatiratanga, in that the basis for both affirming and advocating for rangatiratanga is aimed at collective well-being as opposed to individual well-being. In lieu of face to face interactions, online tools enabled Māori to maintain whanaungatanga through the promotion of specifically Māori material. Māori online webinar series, the development of te reo Māori (Māori language) support, as well as Māori specific business pages all developed during this period which simultaneously advanced Māori knowledge as it did promote whanaungatanga in absence of physical contact. These can all be considered as actions taken to enhance tino rangatiratanga as they were aimed at essentially promoting the well-being of Māori communities through sharing Māori knowledge and Māori expertise.

For example, outside of targeted actions to protect local communities, the Covid-19 alert level restrictions and the resulting limited social interactions led to various online initiatives. Online karakia (prayer), tangihanga (funeral proceedings), research conferences, medical and psychology advice, health messaging, cultural workshops, educational resources, and pages dedicated to supporting Māori businesses through the economic downturn, are just a few of the many examples of the online innovation Māori individuals and communities showed during the lockdown. It is clear from these few examples, that Māori were not only reacting in the space but actively innovating, sharing knowledge, and supporting others.

**Figure 2:**
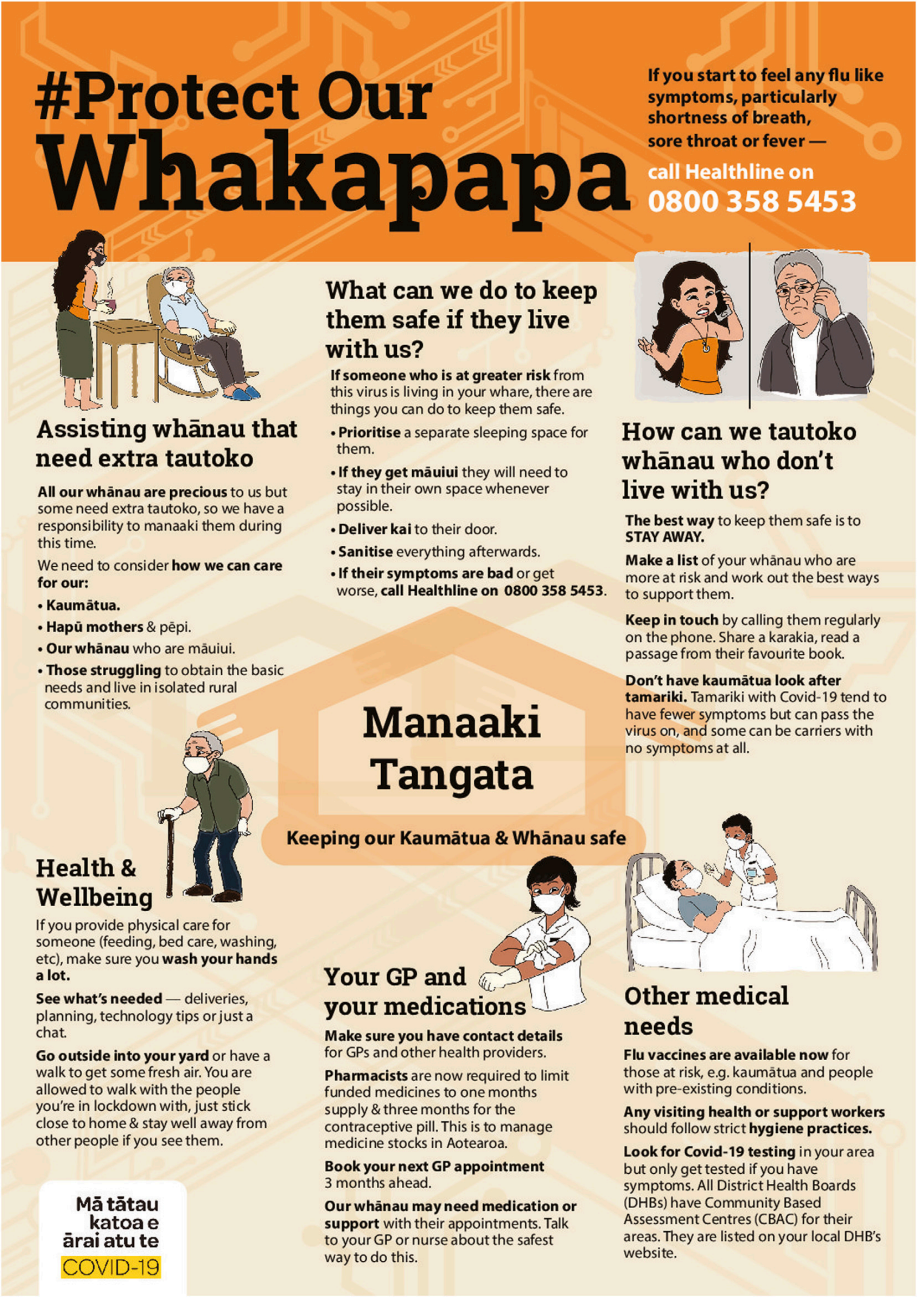
#Protectourwhakapapa Communication Strategy. Source. #ProtectOurWhakapapa (2020).

New grassroots organisations also emerged. During the pandemic, Māori knew that Māori specific health messaging was essential to ensure effective and relevant communication of important health information to Māori communities. Therefore, a grassroots organisation started an online communication strategy and hashtag #Protectourwhakapapa (see Figure 2 for an example) to ensure that there was health communication that was effectively conveyed and relevant to Māori families and communities (McMeeking and Savage, 2020).

Due to Covid-19 restrictions preventing gathering, some normally in-person cultural practices were moved to an online format. This included several people and organisations hosting online karakia (prayer) over Zoom and Facebook, as well as some tangihanga proceedings moving online. While we do not go into significant detail in this article about the process in which these practices were adapted, the important point here is that a significant adaptation to our cultural practices took place. Karakia and even more so tangihanga processes are not common online and indeed in many ways center on face to face interaction. However, Māori recognised the importance of both of these cultural processes for spirituality and emotional health during Covid-19 (as well as the long-term impact of these practises for well-being). Therefore, kaumātua, and those with in-depth knowledge of tikanga and mātauranga Māori, came together to adapt tikanga and create online tangihanga guidelines and also host online karakia. Again this shows the adaptive capacity of our tikanga, when we understand the underlying mātauranga and values guiding our tikanga practices - and this in and of itself demonstrates how tino rangatiratanga and well-being are linked.

These case studies provide examples of how tino rangatiratanga can be enacted through novel and diverse measures which are underpinned by tikanga Māori. In these cases, Māori values and holistic well-being was the key motivation, as Māori responded to the physical, mental, emotional, social, spiritual and economic challenges of Covid-19, in a culturally appropriate manner.

### Holistic Covid-19 Response

The holistic Covid-19 responses worked to ensure that not only were mortality rates low, but also protected wider well-being of Māori communities and in line with the World Health Organization which describes health as a “state of complete physical, mental and social well-being and not merely the absence of disease or infirmity” (World Health Organization, 1995, p. 1). This value-based health approach led to a dynamic and holistic health response by iwi which included consideration of the whole person, taking into account mental, social, and cultural factors, rather than just the absence or presence of the virus.

### Recommendations

The Covid-19 pandemic is still unfolding and presenting the world with unprecedented challenges and questions around how to best manage the ongoing uncertainty and response.From the actions undertaken by Māori and highlighted in this article, there are a number of key lessons that could be implemented to inform future plans. With these case study examples in mind, and in line with a Kaupapa Māori research methodology which suggests that research should lead to transformational change we suggest a number of recommendations for how Aotearoa can plan for a future that enhances both tino rangatiratanga and well-being (Smith, 2013). We offer these simply as options to consider and encourage conversations amongst indigenous and non-indigenous peoples facing this crisis together.

1. Tikanga Māori positioned at the center of decision-making across all political decision-making. Presently, while there are some policies and even fewer laws which mention tikanga Māori, there needs to be greater acceptance of tikanga Māori as key values which can benefit all of Aotearoa New Zealand. As adaptable and flexible values, tikanga have effectively informed key Māori responses to the Covid-19 pandemic.
2. Māori leadership in the responses to Covid-19 and overall health and well-being. This could take the form of an independent Māori health board at the national, regional or local levels, iwi led responses with more decision-making powers. However, whatever the structure that Māori leadership takes, it must be resourced sufficiently and given full control over how resources are used. Several governance groups have already proposed partnership with the government on Covid-19 issues such as the Māori Reference Group, Māori Monitoring Group, Iwi Chairs Forum, and Te Tumu Whakarae. The government has also eventually released a Māori Covid-19 response plan including the formation of a Māori Touchstone Group and almost $NZ50 million in assistance for Māori health providers (Johnsen, 2020), These are good beginnings, however control over Māori health needs to move more rapidly into Māori hands.
3. Supports the establishment of an independent Māori Health Authority which also allows capacity and space for Iwi-specific response, as seen as effective during Covid-19 (Tribunal, 2019).
4. Provide opportunity and resources for Māori to continue to explore the underlying nature of tikanga so that tikanga can continue to inform the future of Aotearoa.
5. Greater collaboration between Māori communities and police. The iwi checkpoints in particular required some level of Māori and police engagement which was largely successful. This was a result of Māori being given support by the police for Māori led initiatives. This could provide a framework for developing Māori and police relationships.
6. 6. Finally, tino rangatiratanga needn’t be considered as a threat. The Covid-19 pandemic has shown that is Māori exercising tino rangatiratanga has in fact led to greater well-being for Māori and non-Māori alike. If encouraged outside of a pandemic, the potential would be even greater.

The Covid response in New Zealand, both by the New Zealand Government and Māori, has highlighted the need for systemic change in health. The case study examples provided in this article and discussion show clearly that Māori need to be given leadership roles in health and well-being plans. While this right to have control over well-being is a central part of tino rangatiratanga, there remain significant challenges around the New Zealand government's willingness to fully accept Māori tino rangatiratanga which is often seen as a threat to governments. However, Covid-19 has and will continue to require innovative and shared responses in order to ensure that the health and well-being of all individuals and groups are protected and enhanced. Māori experience of disease coupled with our continued expressions of tino rangatiratanga, have shown that we are prepared as well as any colonial government, if not more so, to promote the well-being of our communities. Furthermore, the examples have shown how well-being for Māori has been able to thrive in both the numbers of people who contracted Covid-19, as well as well-being that has been enabled through the adaptation of our tikanga to promote social well-being and community learning.

## Conclusion

The New Zealand response to Covid-19 is being heralded internationally as an economic and health success (Cave, 2020). But this success cannot be viewed independently from the Māori Covid-19 response which was even harder and faster than the central government response. Not only did the Māori response likely contribute to the overall New Zealand economic and health success for all New Zealanders but it also provides an example of a more holistic health response - one that not only the New Zealand government but other governments around the world could learn from.

In analysing the Māori response to Covid-19 it is clear that Māori communities reacted with innovative, decisive, and robust decision making, that enacted tino rangatiratanga which was driven in part by tikanga Māori. Furthermore, these Māori philosophical perspectives which resulted in swift and concise actions, demonstrate the close links between tino rangatiratanga and well-being. In other words, each is necessary for the other, and that the health of our communities is dependent on the health of our tino rangatiratanga.

The response to Covid-19 to protect communities and ensure holistic well-being meant for the first time ever, Māori were not disproportionately negatively impacted by an endemic reaching the shores of Aotearoa. We echo the calls from Durie (2020) that have been made clear during this period of time that; *“I would like to see an Aotearoa moving forward, which gives more cognisance to the systems and structures that we have as Māori. What I think we require, is a system in this country which allows us, or gives us more flexibility to do things in a way in which we know work for our people”* (2020).

It is clear that there are parallels across the indigenous community lead responses, who showed strong leadership and a value- driven response to Covid-19. By examining the Covid-19 response in New Zealand, as well as that of other Indigenous peoples around the world, it is clear that Indigenous-led solutions can positively impact the Covid-19 response for all.

## Author Contributions

CC and ATO both made an equal contribution to preparing and writing this manuscript.

## Conflict of Interest

The authors declare that the research was conducted in the absence of any commercial or financial relationships that could be construed as a potential conflict of interest.
